# How ketamine helps to overcome depression

**DOI:** 10.7554/eLife.05418

**Published:** 2014-12-12

**Authors:** Thu N Huynh, Eric Klann

**Affiliations:** Center for Neural Science, New York University, New York, United States; Center for Neural Science, New York University, New York, United Statesek65@nyu.edu

**Keywords:** depression, cortex, synapse, ketamine, electrophysiology, protein synthesis, mouse, rat

## Abstract

Genetically modified mice shed new light on how ketamine can target NMDA receptors in the brain to reduce the symptoms of depression.

**Related research article** Miller OH, Yang L, Wang CC, Hargroder EA, Zhang Y, Delpire E, Hall BJ. 2014. GluN2B-containing NMDA receptors regulate depression-like behavior and are critical for the rapid antidepressant actions of ketamine. *eLife* **3**:e03581. doi: 10.7554/eLife.03581

**Image** The mice lack a subunit of the NMDAR protein in the cortex of the brain (shown in green; scale bar = 1 mm)

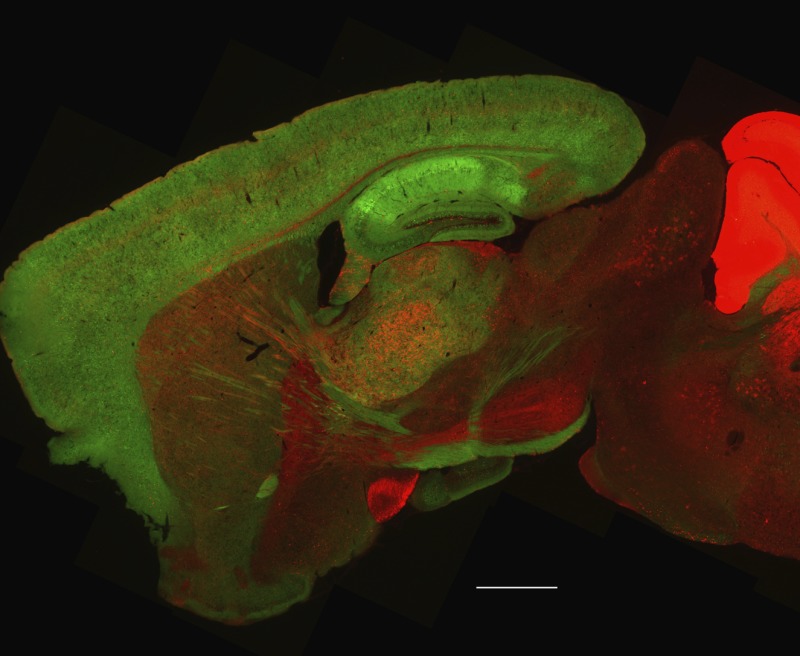

Depression affects hundreds of millions of people and is one of the leading causes of disability worldwide. Unfortunately, current treatments for depression are limited in their efficacy because their therapeutic benefits can take several weeks before setting in and because they are only effective in approximately one third of people with depression. Individuals with treatment-resistant depression often suffer greatly in the absence of an effective drug treatment, which can lead to suicide in severe cases.

Several studies have suggested that ketamine may hold the key to developing a fast-acting antidepressant therapy. Ketamine targets a large protein complex found on the surface of neurons called the N-methyl-D aspartate receptor (NMDAR). Studies have shown that blocking NMDAR proteins with ketamine reverses depressive-like behavior more quickly and more effectively than traditional antidepressant medications (Zarate et al., 2006; Li et al., 2010; Autry et al., 2011). It can also reduce suicidal thoughts in individuals with treatment-resistant depression (DiazGranados et al., 2010). However, the popularity of ketamine as a recreational drug has led to recent research focusing on how it works rather than exploring its potential to treat depression.

One way that ketamine exerts its antidepressant effects is by increasing protein synthesis and increasing excitatory activity (as opposed to inhibitory activity) in neurons (Li et al., 2010). Also, it has similar effects to drugs that specifically block one of the subunits of the NMDAR protein, known as GluN2B (Li et al., 2010). Chemicals that block this subunit were demonstrated to have fast acting antidepressant effects in both humans and rodents (Preskorn et al., 2008; Li et al., 2010). However, not all NMDAR proteins contain this subunit, so it is not clear if drugs that block the GluN2B subunit specifically and ketamine (which blocks all NMDAR proteins) have similar effects on protein synthesis and the excitatory activity of neurons. Now, in eLife, Benjamin Hall and colleagues from Tulane University and Vanderbilt University Medical Center—including Oliver Miller and Lingling Yang as joint first authors—address this question (Miller et al., 2014).

To study how ketamine influences NMDAR proteins, Miller, Yang et al. generated a mouse line (called 2BΔCtx) that lacks the GluN2B subunit in excitatory neurons in the cortex. They assessed the baseline levels of depressive-like characteristics of the 2BΔCtx mice using two tests—the tail suspension test and the forced swim test. In these tests, mice with depressive-like characteristics will spend less time moving compared to wild-type mice: this reflects one of the symptoms of depression in humans known as behavioral despair.

The 2BΔCtx mice spent more time moving than their wild-type counterparts during both tests, suggesting that they have lower levels of depressive-like behavior. In fact, the effect was so strong that ketamine—which increased the amount of time the wild-type mice were moving during the tests—had no impact on the 2BΔCtx mice.

Previous work had shown that ketamine leads to an increase in the transmission of excitatory signals in neurons, so Miller, Yang et al. hypothesized that these changes would be mimicked in the 2BΔCtx mice (Li et al., 2010). Indeed, the 2BΔCtx mice had higher levels of excitatory activity in the prefrontal cortex of the brain, and ketamine—which increased excitatory activity in the wild-type mice—had no additional effect. These results indicate that the antidepressant action of ketamine may be caused by the changes in the excitation of neurons.

The 2BΔCtx mice also allowed Miller, Yang et al. to study how ketamine reduces depressive-like behavior, and how NMDAR subunits are involved, in greater detail. It had been shown previously that ketamine increased the levels of specific proteins in the cortex. However, the overall levels of protein synthesis following ketamine treatment have not been measured (Li et al., 2010). Using a method of protein tagging called FUNCAT—which makes it possible to see when proteins are being made (Dieterich et al., 2010)—they confirmed that there is an increase in the baseline levels of protein synthesis in neurons from 2BΔCtx mice compared to neurons from wild-type mice.

Finally, this work raises the question of how ketamine seems to produce similar effects as chemicals that specifically target the GluN2B subunit. The complete NMDAR protein is made of four subunits that fit together to form a channel across the cell membrane. The neurotransmitter glutamate activates NMDAR proteins via the GluN2B subunit to open the channel and allow positively charged ions to pass through the membrane, which leads to a series of events that inhibit protein synthesis. The FUNCAT data shows that NMDAR proteins containing a GluN2B subunit are constantly active: Miller, Yang et al. suggest that this is due to the low levels of glutamate found in the neurons (Figure 1) (Meldrum, 2000). By treating 2BΔCtx mice with several different chemicals, they elegantly showed that the flow of positive ions across the membrane generated by low levels of glutamate was due to NMDAR proteins that contained GluN2B subunits.

**Figure 1.**
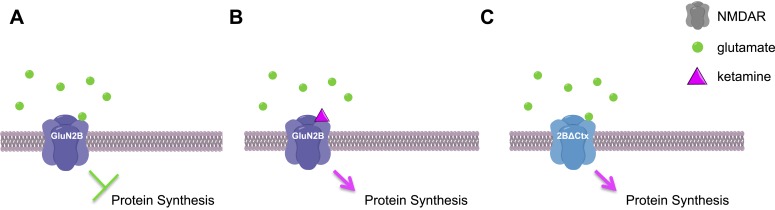
Ketamine increases protein synthesis in neurons and decreases depressive-like behaviors. (**A**) In the neurons of wild-type mice, low levels of glutamate (green circles) activate NMDAR proteins that contain GluN2B subunits, leading to a decrease in protein synthesis and an increase in depressive-like behaviors. (**B**) If ketamine (purple triangle) is administered to these mice it blocks the NMDAR proteins, leading to an increase in protein synthesis and a decrease in depressive-like behaviors. (**C**) The NMDAR proteins in 2BΔCtx mice—which lack the GluN2B subunit in cortical neurons—cannot be activated by glutamate, so there is an increase in protein synthesis and a decrease in depressive-like behaviors, even in the absence of ketamine. This image was generated using ChemDraw.

Harnessing the properties of ketamine into a more specific drug therapy has been a goal of many researchers since the discovery of ketamine as a fast-acting antidepressant. Miller, Yang et al. have made a huge leap in helping to uncover exactly how ketamine may be working. Although drugs that target the GluN2B subunit also have been reported to have antidepressant properties, this latest work significantly expands on the previous literature confirming that the GluN2B subunits are viable therapeutic targets for depression.
